# Mutational spectrum of p53 gene in arsenic-related skin cancers from the blackfoot disease endemic area of Taiwan

**DOI:** 10.1038/sj.bjc.6690467

**Published:** 1999-06

**Authors:** C-H Hsu, S-A Yang, J-Y Wang, H-S Yu, S-R Lin

**Affiliations:** Departments of Dermatology; Clinical Pathology and; Surgery, Kaohsiung Medical College, No. 100, Shih-Chuan 1st Road, Kaohsiung 80317, Taiwan

**Keywords:** p53 gene, arsenic-related skin cancers

## Abstract

To understand the role of p53 tumour suppressor gene in the carcinogenesis of arsenic-related skin cancers from the blackfoot disease endemic area of Taiwan, we collected tumour samples from 23 patients with Bowen's disease, seven patients with basal cell carcinomas (BCC) and nine patients with squamous cell carcinomas (SCC). The result showed that p53 gene mutations were found in 39% of cases with Bowen's disease (9/23), 28.6% of cases with BCC (2/7) and 55.6% of cases with SCC (5/9). Most of the mutation sites were located on exon 5 and exon 8. Moreover, the results from direct sequencing indicated that missense mutations were found at codon 149 (C→T) in one case, codon 175 (G→A) in three cases, codon 273 (G→C) in three cases, codon 292 (T→A) in one case, codon 283 (G→T) in one case, codon 172 (T→C) in one case and codon 284 (C→A) in one case. In addition, silent mutations were also found in four cases. These mutations were located at codons 174, 253, 289 and 298 respectively. In immunohistochemistry analysis, p53 overexpression was found in 43.5% (10/23) of cases with Bowen's disease, 14% (1/7) of cases with BCC and 44% (4/9) of cases with SSC. These findings showed that p53 gene mutation rate in arsenic-related skin cancers from the blackfoot disease endemic area of Taiwan is high and that the mutation types are different from those in UV-induced skin cancers. © 1999 Cancer Research Campaign


*British Journal of Cancer* (1999) **80**(7), 1080–1086
© 1999 Cancer Research Campaign
Article no. bjoc.1998.0467

### Mutational spectrum of p53 gene in arsenic-related skin cancers from the blackfoot disease endemic area of Taiwan

**C-H Hsu[2], S-A Yang[1], J-Y Wang[3], H-S Yu[1] and S-R Lin[2]**

Departments of [1]Dermatology, [2]Clinical Pathology and [3]Surgery, Kaohsiung Medical College, No. 100, Shih-Chuan 1st Road, Kaohsiung 80317, Taiwan

**Summary** To understand the role of p53 tumour suppressor gene in the carcinogenesis of arsenic-related skin cancers from the blackfoot disease endemic area of Taiwan, we collected tumour samples from 23 patients with Bowen's disease, seven patients with basal cell carcinomas (BCC) and nine patients with squamous cell carcinomas (SCC). The result showed that p53 gene mutations were found in 39% of cases with Bowen's disease (9/23), 28.6% of cases with BCC (2/7) and 55.6% of cases with SCC (5/9). Most of the mutation sites were located on exon 5 and exon 8. Moreover, the results from direct sequencing indicated that missense mutations were found at codon 149 (C→T) in one case, codon 175 (G→A) in three cases, codon 273 (G→C) in three cases, codon 292 (T→A) in one case, codon 283 (G→T) in one case, codon 172 (T→C) in one case and codon 284 (C→A) in one case. In addition, silent mutations were also found in four cases. These mutations were located at codons 174, 253, 289 and 298 respectively. In immunohistochemistry analysis, p53 overexpression was found in 43.5% (10/23) of cases with Bowen's disease, 14% (1/7) of cases with BCC and 44% (4/9) of cases with SSC. These findings showed that p53 gene mutation rate in arsenic-related skin cancers from the blackfoot disease endemic area of Taiwan is high and that the mutation types are different from those in UV-induced skin cancers.

**Keywords:** p53 gene; arsenic-related skin cancers

Blackfoot disease is a peripheral vascular disease resulting in gangrene of the lower extremities (Pan et al, 1993). The endemic area is along the south-west coast in Taiwan (Tseng et al, 1968; Brown et al, 1995; Tseng et al, 1995). People living in the endemic area have a higher rate of skin, bladder, renal and lung cancers than those in other areas (Chiang et al, 1993; Shibata et al, 1994). Numerous studies have indicated that high levels of arsenic and fluorescent substances are present in artesian well water of the endemic area (Chen et al, 1988; Wu et al, 1989; Chiou et al, 1995; Hsueh et al, 1995). Arsenic contamination of drinking water is known to cause chronic poisoning and an association between the effects of chronic exposure to a high level of arsenic in drinking water and the occurrence of a variety of skin disorders, skin cancer and blacfoot disease was found in 1968 (Tseng et al, 1968). Recently, carcinogenesis has been studied using molecular biological methods. Carcinogenesis has been suggested as a multistep process that occurs in a whole animal and needs multiple gene alterations (Bishop et al, 1991). Some genes, for example p53, have been found to be involved in carcinogenesis. In ultraviolet (UV)-induced skin cancer Oram et al (1994) found that p53 tumour suppressor gene mutation rate was 50%; a similar mutation rate was found by other authors (Brash et al, 1991; Sato et al, 1993; Ziegler et al, 1993; Matsumura et al, 1996). Most of the mutation types are CC→TT transitions (Lubbe et al, 1994; Nakazawa et al, 1994; Ziegler et al, 1994; Drouin et al, 1997). The carcinogenesis of arsenic-related skin cancer, however, is very different from that of UV-induced skin cancer. To understand the role of p53 tumour suppressor gene in arsenic-related skin cancer, we analysed p53 gene mutation in 39 cases with non-melanoma skin cancer from the blackfoot disease endemic area. Furthermore, we compared the carcinogenic mechanism of arsenic-related cancers with that of UV-induced skin cancer.

*Received 27 April 1998*
*Revised 17 November 1998*
*Accepted 23 November 1998*

*Correspondence to:* S-R Lin

### MATERIALS AND METHODS

### Specimen collections

All of the tumour tissue specimens of arsenic-related skin cancer and normal tissue specimens were collected from patients living in the blackfoot disease endemic area of Taiwan. The patients included 23 patients with Bowen's disease, seven patients with basal cell carcinomas (BCC) and nine patients with squamous cell carcinomas (SCC); 15 of the patients also had blackfoot disease. All of the tumour specimens were collected from areas not generally exposed to the sun, including the inner thigh, upper back, loin, interior upper arm and penis. In addition, the pathological biopsy of the cancers did not reveal solar elastosis, which is caused by sun damage (Weyers et al, 1996). The specimens were partially formalin-fixed and wax-embedded. The other unfixed tissues were fresh frozen in liquid nitrogen for DNA extraction.

### DNA extraction

Genomic DNA was extracted from tissues by proteinase K digestion and phenol–chloroform extraction according to Sambrook's method (Sambrook et al, 1989).

**Table 1** Results of p53 mutations in 39 cases with arsenic-related skin cancer

| Case no. | Sex | Age | Diagnosis | Site | No. of lesion | SSCP | Exon | Codon | a.a. base | IMH | BFD |
|---|---|---|---|---|---|---|---|---|---|---|---|
| 1 | M | 57 | BD | Upper back | 3 | N | | | | – | – |
| 2 | M | 61 | BD | Loin (Lumbar) | 3 | N | | | | – | – |
| 3 | M | 64 | BD | Interior upper arm | 5 | M | 7 | 249, AGG→AAG | Arg→Lys | + | + |
| 4 | M | 58 | BD | Loin (Lumbar) | 2 | M | 8 | 292, AAA→AAT | Lys→Asn | + | – |
| 5 | M | 63 | BD | Penis | 1 | N | | | | – | – |
| 6 | M | 67 | BD | Interior thigh | 3 | M | 5 | 149, TCC→TTC | Ser→Phe | + | + |
| 7 | M | 69 | BD | Abdomen | 4 | N | | | | – | – |
| 8 | M | 54 | BD | Abdomen | 3 | N | | | | + | – |
| 9 | F | 68 | BD | Interior upper arm | 3 | N | | | | – | – |
| 10 | F | 53 | BD | Neck | 1 | M | 8 | 273, CGT→CCT | Arg→Pro | + | + |
| 11 | F | 73 | BD | Lower back | 3 | M | 5 | 175, CGC→CAC | Arg→His | + | + |
| 12 | F | 64 | BD | Forehead | 1 | N | | | | + | – |
| 13 | F | 69 | BD | Upper back | 4 | N | | | | – | – |
| 14 | F | 65 | BD | Palm | 1 | N | | | | – | – |
| 15 | M | 72 | BD | Interior thigh | 3 | M | 8 | 298, GAG→GAA | Silent | – | – |
| 16 | M | 53 | BD | Abdomen | 4 | M | 5 | 175, CGC→CAC | Arg→His | + | + |
| 17 | F | 60 | BD | Lower back | 2 | N | | | | – | – |
| 18 | F | 65 | BD | Lower back | 4 | N | | | | + | – |
| 19 | M | 68 | BD | Interior upper arm | 3 | N | | | | – | – |
| 20 | M | 69 | BD | Abdomen | 3 | M | 7 | 253, ACC→ACG | Silent | – | – |
| 21 | F | 67 | BD | Lower back | 2 | N | | | | – | – |
| 22 | M | 53 | BD | Interior thigh | 1 | M | 5 | 175, CGC→CAC | Arg→His | + | + |
| 23 | F | 64 | BD | Loin | 2 | N | | | | – | – |
| 24 | F | 73 | BCC | Interior thigh | 1 | M | 5 | 174, AGG→CGG | Silent | – | + |
| 25 | M | 67 | BCC | Abdomen | 1 | N | | | | – | – |
| 26 | F | 58 | BCC | Front of elbow | 2 | N | | | | – | + |
| 27 | F | 62 | BCC | Interior upper arm | 1 | N | | | | – | – |
| 28 | M | 71 | BCC | Upper back | 2 | N | | | | – | – |
| 29 | M | 73 | BCC | Loin (Lumber) | 3 | N | | | | – | + |
| 30 | M | 71 | BCC | Lower back | 2 | M | 8 | 283, CGC→CTC | Arg→Leu | + | + |
| 31 | F | 60 | SCC | Lower back | 1 | M | 5 | 172, GTT→GCT | Val→Ala | – | + |
| 32 | M | 56 | SCC | Interior thigh | 1 | N | | | | + | + |
| 33 | F | 67 | SCC | Penis | 1 | N | | | | – | – |
| 34 | M | 61 | SCC | Lower back | 2 | M | 8 | 273, CGT→CCT | Arg→Pro | + | + |
| 35 | F | 73 | SCC | Abdomen | 1 | N | | | | – | – |
| 36 | M | 59 | SCC | Abdomen | 2 | M | 8 | 284, ACA→AAA | Thr→Lys | + | + |
| 37 | M | 64 | SCC | Upper back | 2 | M | 8 | 273, CGT→CCT | Arg→Pro | + | + |
| 38 | F | 68 | SCC | Penis | 1 | N | | | | – | – |
| 39 | M | 58 | SCC | Chest | 2 | M | 8 | 289, CTC→CTG | Silent | – | – |

IMH: immunohistochemical staining; BFD: blackfoot disease.

### PCR-SSCP analysis

To search for subtle mutation of the p53 gene using polymerase chain reaction–single-strand conformation polymorphism (PCR-SSCP) analysis, 11 different sets of primers for p53 coding regions were used and are described below:

Exon 2: SR23LT: 5′-AGCCAGACTGCCTTCCGGGTCA-3′; SR23RT: 5′-TGGCATTCTGGGAGCTTC-3′; Exon 4: SR4-1LT: 5′-TGCCGTCCCAAGCAATGGAT-3′; SR4-1RT: 5′-CTGGGAAGGGACAGAAGATGA-3′; SR4-2LT: 5′-GTGGC-CCCTGCACCAGCAGCT-3′; SR4-2RT: 5′-CTCAGGGCTTCTGACCGTGCA-3′; Exon 5: SR5-1LT: 5′-CTTTGCTGCCGTCTTCCAGTTCG-3′; SR5-1RT: 5′-CTAT-CTGAGCAGCGCTCATG-3′; SR5-2LT: 5′-GCCATCTACAAGCAGTCA-3′; SR5-2RT: 5′-AGACC-TAAGAGCAATGAGTG-3′; Exon 6: SR6LT: 5′-AGGTCTG-GCCCCTCCTCAGC-3′; SR6RT: 5′-ACCTCAGGCGGCTCATAGGGCA-3′; Exon 7: SR7LT: 5′-TCTCCTAGGTTGGCTCTGAC-3′; SR7RT: 5′-CACAGCAGGCCAGTGTGCAG-3′ Exon 8: SR8LT: 5′-TGGGACAGGTAGGACCTGA-3′; SR8RT: 5′-TGAATCTGAGGCATAACTGCACC-3′; Exon 9: SR9LT: 5′-GGAGCACCGCAGGGTGCAG-3′; SR9RT: 5′-CCCAA-GACTTAGTACCTGAA-3′; Exon 10: SR10LT: 5′-CTCTGTTGCTGCAGATC-3′; SR10RT: 5′-GCTGAGGT-CACTCACCT-3′; Exon 11: SR11LT: 5′-GAATTCTGTCTCC-TACAGCCAC-3′; SR11RT: 5′-GAATTCTGACGCACAGGTATTGC-3′.

The reaction mixture contained 50 pmol of each primer, 2.5 U *Taq* DNA polymerase (Promega Corp., Madison, WI, USA), 100 mmol/l$^{-1}$ of each deoxy-NTP, [α-$^{32}$P]deoxy-CTP (3000 Ci mmol$^{-1}$; 10 Ci ml$^{-1}$; New England Nuclear Research Products, Boston, MA, USA), 1.5 mmol l$^{-1}$ magnesium chloride, 50 mmol l$^{-1}$ potassium chloride, 10 mmol l$^{-1}$ Tris–HCl pH 8.3 and gelatin at 10 μg ml$^{-1}$. A programmable thermal cycler (PTC-100, MJ Research, Watertown, MA, USA) was used to perform 40 cycles of for exons 2, 3, 4, 5, 6, 7, 10 and 11, at 58°C for exon 8, and at 62°C for exon 9 with an extension for an additional 2 min at 72°C. Re-PCR of mutated DNA was done to ensure the reproducibility.

*© 1999 Cancer Research Campaign*

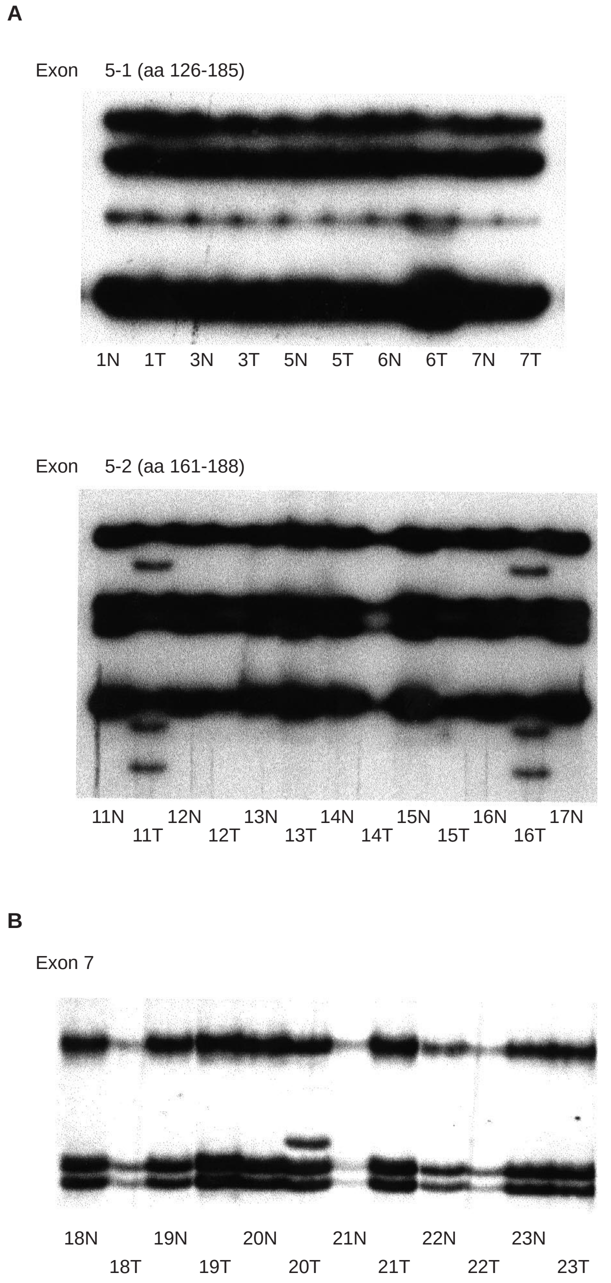

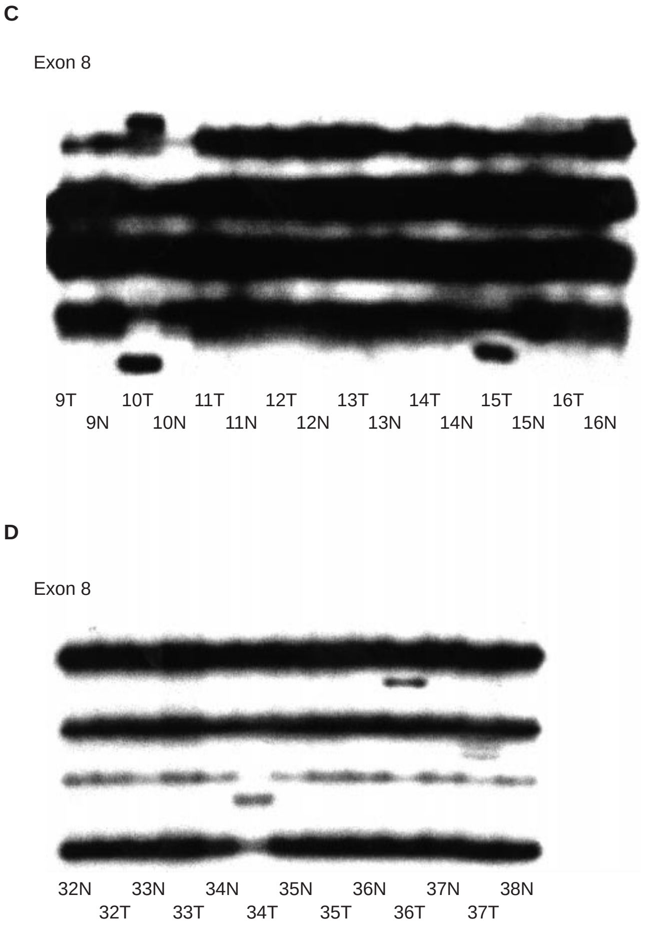

**Figure 1** PCR-SSCP analysis of p53 mutations in arsenic-related skin cancers. Representative samples are shown for exons 5 (**A**), 7 (**B**) and 8 (**C, D**). An electrophoretic mobility shift of the bands differs between the tumour (T) and its paired normal tissues (N), representing a different conformer of the fragment and suggesting the presence of mutations in these exons. The representative normal PCR-SSCP results of exon 7 are shown in (**C**). Note, no electrophoretic mobility shift over the samples tested suggests normal conformations over these samples

### Direct sequencing

DNA extracts for DNA sequencing were amplified using the primers SR4-2LT and SR5-1RT for the p53 exon 5 region, and using the primers SR6LT and SR8RT for the p53 exon 8 region. The PCR products were furthermore purified by Chroma spin column (Clontech Laboratories) and then subjected to sequencing using a double-strand cycle sequencing system (BRL, Life Technologies, Inc.) with the primer SR5LT for exon 5 and SR8LT for exon 8 (Innis et al, 1988).

### Immunohistochemistry

Tissue samples were mounted in tissue-Tek OCT compound (Ames Division, Miles Laboratory, Elkhart, IN, USA), and 8-μm sections of each skin cancer specimen were cut, air-dried and fixed in acetone for 10 min. Immunological detection was performed using anti-p53 monoclonal antibody pAbDO-1 (Oncogene Science, Inc., Manhasset, NY, USA), which recognizes both wild-type and mutant forms of the p53 protein. The binding of the antibody was visualized by the avidin–biotin complex peroxidase technique, using a sensitive Vectastain Elite kit (Vector Laboratories, Burlingame, CA, USA) immunoperoxidase system.

### RESULTS

Thirty-nine tumour samples and normal tissue samples were collected from 23 patients with Bowen's disease, seven patients with BCC and nine patients with SCC. Genomic DNA was

*© 1999 Cancer Research Campaign*

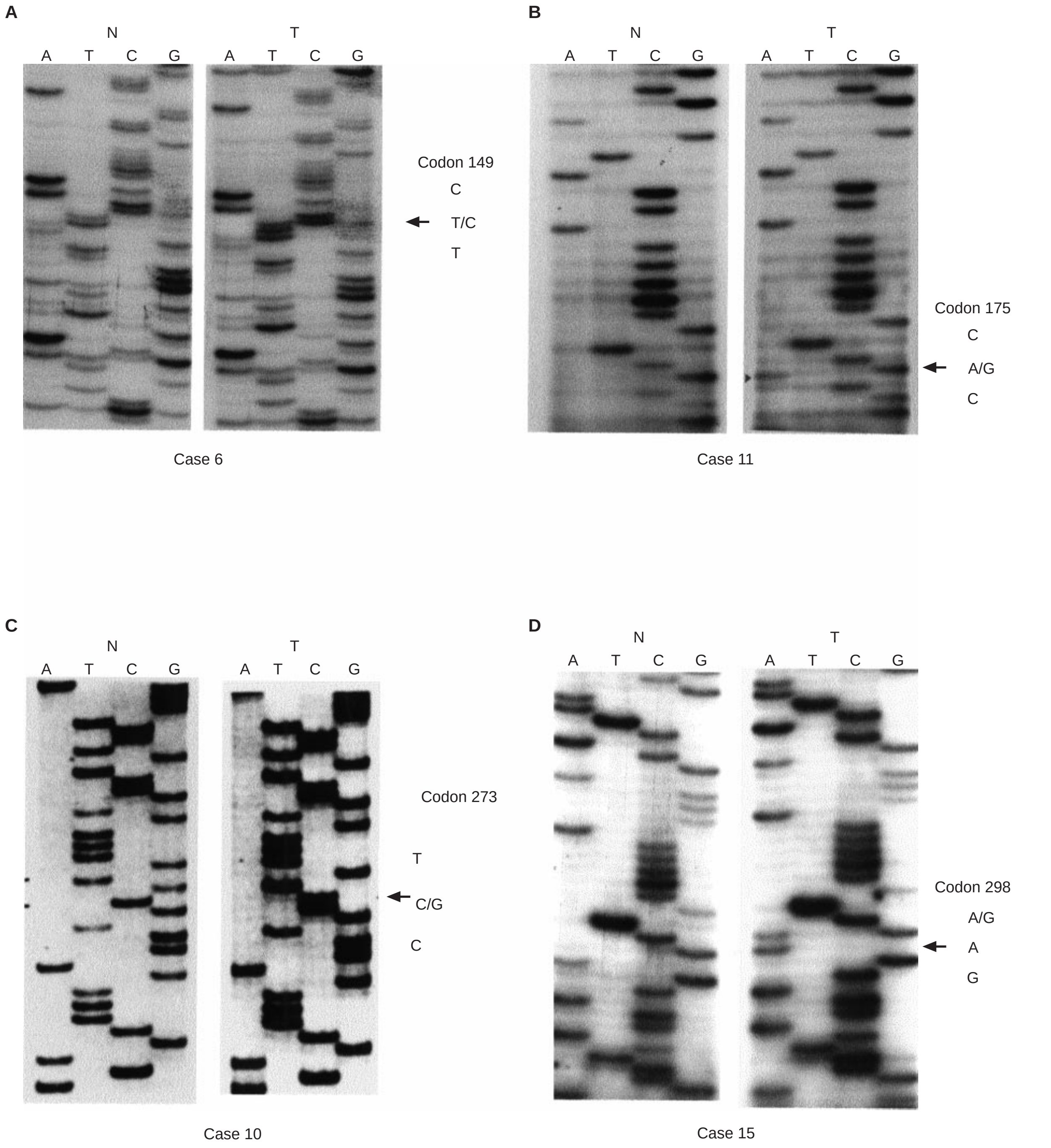

**Figure 2** Nucleotide sequencing analysis of the mutations in arsenic-related skin cancers. Each mutation in the tumour cells (T) shown is matched to a normal adjacent skin tissue (N). The codon at which the mutation occurs is indicated. Each sequence is shown 5′ (bottom) to 3′ (top). Arrows point to bands corresponding to mutated basepairs. (**A**) Sequencing gel shows a p53 point mutation at codon 149 in tumour cells of case 6 resulting in a change in the encoding amino acid from serine to phenylalanine. (**B**) A p53 point mutation at codon 175 in tumour cells of case 11 results in a change in the encoding amino acid from arginine to histidine. (**C**) Sequencing gel reveals a point mutation at codon 273 in the tumour cells of case 10, results in a change in the encoding amino acid from arginine to proline. (**D**) A point mutation at codon 298 in the tumour cells of case 15 resulting in a silent mutation extracted from the tumour and normal tissues and then PCR-SSCP was performed to screen the alterations of p53 gene from exon 2 to exon 11. The results are summarized in Table 1. After neutral polyacrylamide gel electrophoresis, the analysis of exon 7 showed mobility shift bands in cases 3 and 20 (Figure 1B); the analysis of exon 5 showed mobility shift bands in cases 6, 11, 16, 22, 24 and 31, in addition to the normal bands (Figure 1A). Moreover, the SSCP patterns revealed that the mobility shift patterns of cases 11, 16 and 22 were the same. In analysis of exon 8, the mobility shift was also observed in cases 4, 10, 15, 30, 34, 36, 37 and 39 (Figure 1-C, D). No significant DNA mobility shift was observed in the analysis of other exons (data not shown).

PCR products displaying a mobility shift on SSCP analysis were directly sequenced. The results of direct sequencing were compared

*© 1999 Cancer Research Campaign*

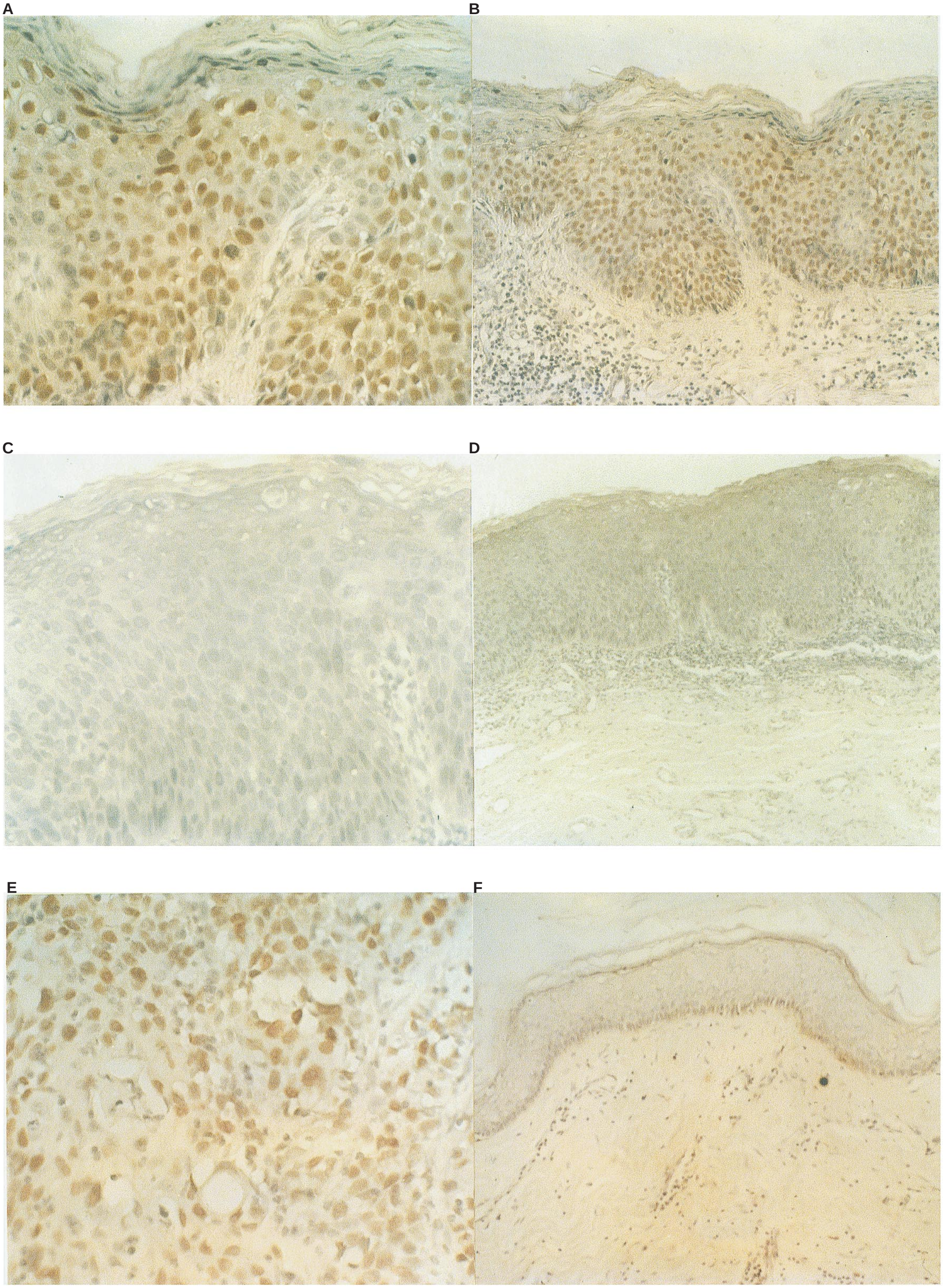

**Figure 3** Immunohistochemical studies of p53 protein in arsenic-related skin cancers. Cryostat sections were fixed in acetone and stained with anti-p53 monoclonal antibody pAbDO-1 and detected by the peroxidase–antiperoxidase method. The studies were counterstained with haematoxylin. Positive staining was found over the skin cancer cells of a representative case with Bowen's disease (**A**, × 500; **B**, × 250). Note the intense nuclear brownish staining in the majority of tumour cells limited to the superficial areas. The brownish staining was the result of enzyme reaction with substrate diaminobenzidine, and absence of staining over the control section occurred when the primary antibody was replaced with non-immune antiserum (**C**, × 500; **D**, × 250). Overexpression of p53 protein was also noticed in the pathological sections of SCC patients (**E**, × 500). Note the brownish intranuclear staining found in the cancer cells passed the basal membrane and infiltrated in the dermis (**F**, × 250). The immunohistochemical results of a normal skin tissue stain with pAbDO-1 monoclonal antibody. No obvious nuclear staining was observed over the whole area, indicating that normal p53 level in the normal skin tissue is rather low

 *© 1999 Cancer Research Campaign*

to those of the controls. The results indicated that the mutation sites in exon 5 were found to be at codon 149 (TCC→TTC) in one case (Figure 2A), resulting in a change from serine to phenylalanine in the protein; at codon 175 (CGC→CAC) in three cases, resulting in a change from arginine to histidine (Figure 2B); at codon 174 (AGG→CGG) in one case, resulting in a silent mutation; and at codon 172 (GTT→GCT) in one case, resulting in a change from valine to alanine in the protein. The mutation sites in exon 7 were found to be located at codon 249 (AGG→AAG) in one case, leading to a substitution of lysine for arginine in the protein, and at codon 253 (ACC→ACG) in one case, leading to a silent mutation. In addition, the mutation sites in exon 8 were found to be at codon 292 (AAA→AAT) in one case, leading to a substitution of asparagine for lysine in the protein; at codon 273 (CGT→CCT) in three cases (Figure 2C), leading to a substitution of proline for arginine in the protein; at codon 284 (ACA→AAA) in one case, resulting in a change from threonine to lysine and at codon 298 (GAG→GAA) in one case; and at codon 289 (CTC→CCT) in one case, leading to a silent mutation (Figure 2D).

In immunohistochemical staining analysis, ten of 23 cases with Bowen's disease showed that their tumour cells were limited to the epidermis. The tumour cells displayed predominantly brown nuclear staining, indicating the overexpression of the p53 protein (Figure 3A, B). On the other hand, the pathological sections of SCC patients revealed that the cancer cells passed the basal membrane and infiltrated to the dermis, also indicating the overexpression of the p53 protein (Figure 3E).

### DISCUSSION

The tumour samples we collected for this study were from residents in the blackfoot disease endemic area of Taiwan where the arsenic content in the well water is reportedly high. Several statistical analyses showed that the residents who live in the blackfoot disease endemic area have a higher arsenic species content in urine and a higher occurrence of skin, bladder and lung cancers than those who live in other areas of Taiwan (Tseng et al, 1968; Chiang et al, 1993; Hsueh et al, 1998). Arsenic is known to influence cell division (Debec et al, 1990), and increases chromosomal breaks, sister chromatid exchange and morphological transformation in mammalian cells (Magos et al, 1991). Numerous studies on cultured cell and animal models have shown that arsenic may generate reactive oxygen species to exert its toxicity (Lee-Chen et al, 1993; Cavigelli et al, 1996; Lynn et al, 1998), enhance gene amplification and inhibit DNA repair process (Lee et al, 1988). Shibata et al suggested that inorganic arsenic compounds act as co-carcinogens rather than primary carcinogens in vivo as well as in vitro (Shibata et al, 1994). The arsenic-induced cellular changes may help increase our understanding of human carcinogenesis.

The results of sequencing showed six types of p53 mutations. One of the types was G:C→A:T transition. The mutation rate was 38%. These results agreed with previous published results of the analysis of p53 alterations in bladder cancer from the blackfoot disease endemic area in Taiwan (Shibata et al, 1994), but were much lower than seen in UV-induced skin cancers (Sato et al, 1993; Inga, 1998). However, we could not neglect the possibility that UV may be involved in the cancer development. The mutation hot spot (codon 175) found in three cases in our study was the same hot spot found in Shibata et al, (1994) study. Additionally, the hot spot has been found to be rare in bladder cancers from other areas and in UV-induced skin cancers (Ziegler et al, 1993; Shibata et al, 1994). The other mutation types (A:T→T:A, G:C→C:G, G:C→T:A, and A:T→C:G) are related to chemical damage of DNA (Matsumura et al, 1996). Furthermore, we compared our results with the results of UV-induced skin cancer done by Nakazawa et al (1994) and Ziegler et al (1993). The occurrence of two types of mutations (C→T or CC→TT) which were predominantly found in UV-induced DNA damage were compared using $\chi^2$ analysis and Fisher's exact test. Significant difference was found between UV-induced skin cancers and arsenic-related skin cancers ($P < 0.05$). These results suggest that UV mutations have little impact on carcinogenesis. Therefore, arsenic exposure may be a major cause of the cancer.

In analysis of p53 protein overexpression, we observed positive staining in 43.5% (10/23) of the cases with Bowen's disease and 55.6% (4/9) of the SCC patients. The prevalence of p53 protein overexpression in UV-induced skin cancer ranged from 20% to 80% in previous reports (Nagano et al, 1993; van Kranen et al, 1995, 1997; Berg et al, 1996; Nataraj et al, 1996). The significant positive rate in Bowen's disease patients suggested that p53 gene mutation commonly occurred in the early stages of arsenic-related skin cancer. PCR-SSCP analysis did not detect p53 gene alterations in four of 15 immunohistochemically stained positive cases. This result may be due to the detection limitation of PCR-SSCP, or the mutation occurring on an intron where it was not detected. p53 protein overexpression was not found in five of 15 cases with p53 gene mutations; this may be due to the mutation belonging to silent mutation or missense mutation, which does not affect the half-life of p53 protein (Lin et al, 1994). The results of this study indicated that the p53 gene mutation rates, sites and types in arsenic-related skin cancer were significantly different from those in UV-induced skin cancer. This proved that, at the molecular level, the cancers occurred at the same portions, but were induced by different carcinogens, and that the p53 gene mutation was associated with the carcinogenesis of arsenic-related skin cancers.

### ACKNOWLEDGEMENTS

This work was supported by a grant from the National Science Council of the Republic of China (NSC-82-0115-B037-114).

### REFERENCES

Berg RJ, van Kranen HJ, Rebel HG, de Vries A, van Vloten WA, Van Kreijl CF, van der Leun JC and de Gruijl FR (1996) Early p53 alteration in mouse skin carcinogenesis by UVB radiation: immunohistochemical detection of mutant p53 protein in clusters of preneoplastic epidermal cells. *Proc Natl Acad Sci USA* **93**: 274–278

Bishop JM (1991) Molecular themes in oncogenesis. *Cell* **64**: 235–248

Brash DE, Rudolph JA, Simon JA, Lin A, McKenna GJ, Baden HP, Halperin AJ and Ponten J (1991) A role for sunlight in skin cancer: UV-induced p53 mutations in squamous cell carcinoma. *Proc Natl Acad Sci USA* **88**: 1012–1018

Brown KG and Chen CJ (1995) Significance of exposure assessment to analysis of cancer risk from inorganic arsenic in drinking water in Taiwan. *Risk Anal* **15**: 475–484

Cavigelli M, Li WW, Lin A, Su B, Yoshioka K and Karin M (1996) The tumor promoter arsenite stimulates AP-1 activity by inhibiting JNK phosphatase. *EMBO J* **15**: 6269–6279

Chen CJ, Wu M, Lee SS, Wang JD, Cheng SH and Wu HY (1988) Atherogenicity and carcinogenicity of high-arsenic artesian well water. Multiple risk factors and related malignant neoplasms of blackfoot disease. *Arteriosclerosis* **8**: 452–460

Chiang HA, Guo HR, Hong CL, Lin SM and Lee EF (1993) The incidence of bladder cancer in black foot disease endemic area in Taiwan. *Br J Urol* **71**: 274–278

© 1999 Cancer Research Campaign 

Chiou HY, Hsueh YM, Liaw KF, Horng SF, Chiang MH, Pu YS, Lin JS, Huang CH and Chen CJ (1995) Incidence of internal cancers and ingested inorganic arsenic: a seven-year follow-up study in Taiwan. *Cancer Res* **55**: 1296–1300

Debec C, Courgeon AM, Mainground M and Maisonhaute C (1990) The response of the centrosome to heat shock and related stresses in a Drosophila cell line. *J Cell Sci* **96**: 403–412

Drouin R and Therrien JP (1997) UVB-induced cyclobutane pyrimidine dimer frequency correlates with skin cancer mutational hotspots in p53. *Protochem Photobiol* **66**: 719–726

Hsueh YM, Cheng GS, Wu MM, Yu HS, Kuo TL and Chen CJ (1995) Multiple risk factors associated with arsenic-induced skin cancer, effects of chronic liver disease and malnutritional status. *Br J Cancer* **7**: 109–114

Hsueh YM, Huang CC, Wu WL, Chen HM, Yang MH, Lue LC and Chen CJ (1998) Urinary levels of inorganic and organic arsenic metabolites among residents in arseniasis-hyperendemic area in Taiwan. *J Toxicol Environ Health* **54**: 431–444

Inga A, Scott G, Monti P, Aprile A, Abbondandolo A, Burns PA and Fronza G (1998) Ultraviolet-light induced p53 mutational spectrum in yeast is indistinguishable from p53 mutations in human skin cancer. *Carcinogenesis* **19**: 741–746

Innis MA, Myambo KB, Gelfand DH, Brow MA, Tseng WP, Chu HM, How SW, Fong JM, Lin CS and Yeh S (1988) DNA sequencing with Thermus-aquaticus DNA polymerase and direct sequencing of polymerase chain reaction-amplified DNA. *Proc Natl Acad Sci USA* **85**: 9436

Lee TC, Takata N, Lamb PW, Gilmer TM and Barrett JC (1988) Induction of gene amplification by arsenic. *Science* **241**: 79–81

Lee-Chen SF, Gurr JR and Jan KY (1993) Arsenite enhances DNA double-strand breaks and cell killing of methyl methanesulfonate-treated cells by inhibiting the excision of alkali-labile sites. *Mutat Res* **294**: 21–28

Lin SR, Lee YJ and Tsai JH (1994) Mutations of the p53 gene in human functional adrenal neoplasms. *J Clin Endocrin Metab* **78**: 483–491

Lubbe J, Kleihues P and Burg G (1994) The tumor suppression gene p53 and its significance for dermatology. *Hautarzt* **45**: 741–745

Lynn S, Shiung JN, Gurr JR and Jan KY (1998) Arsenite stimulates poly (ADP-ribosylation) by generation of nitric oxide. *Free Biol Med* **24**: 442–449

Magos L (1991) Epidemiological and experimental aspects of metal carcinogenesis: physicochemical properties, kinetics, and active species. *Environ Health Perspect* **95**: 157–189

Matsumura Y, Nishigori C, Yagi T, Imamura S and Takebe H (1996) Characterization of p53 gene mutations in basal-cell carcinomas: comparison between sun-exposed and less-exposed skin areas. *Int J Cancer* **65**: 778–780

Nakazawa H, English D, Randell PL, Nakazawa K, Martel N, Armstrong BK and Yamasaki H (1994) UV and skin cancer specific p53 gene mutation in normal skin as a biologically relevant exposure measurement. *Proc Natl Acad Sci USA* **91**: 360–364

Nagano T, Ueda M and Ichihashi M (1993) Expression of p53 protein is an early event in ultraviolet light-induced cutaneous squamous cell carcinogenesis. *Arch Dermatol* **129**: 1157–1161

Nataraj AJ, Black HS and Ananthaswamy HN (1996) Signature p53 mutation at DNA cross-linking sites in 8-methoxypsoralen and ultraviolet A (PUVA)-induced murine skin cancers. *Proc Natl Acad Sci USA* **93**: 7961–7965

Oram Y, Orengo I, Baer SC and Ocal T (1994) p53 protein expression in squamous cell carcinomas from sun-exposed and non-sun-exposed sites. *J Am Acad Dermatol* **31**: 417–422

Pan TC, Horng CJ, Lin SR, Lin TH and Huang CW (1993) Simultaneous determination of Zn, Cd, Pb, and Cu in urine of patients with blackfoot disease using anodic stripping voltammetry. *Biol Trace Elem Res* **38**: 233–241

Sambrook J, Fritsch EF and Maniatis T (1989) *Molecular Cloning*, 2nd edn, *A Laboratory Manual*. Cold Spring Harbor Laboratory: New York.

Sato M, Nishigori C, Zghal M, Yagi T and Takebe H (1993) Ultraviolet-specific mutations in p53 gene in skin tumors in Xeroderma pigmentosum patients. *Cancer Res* **53**: 2944–2946

Shibata A, Ohneseit PF, Tsai YC, Spruck CH III, Nichols PW, Chiang HS, Lai MK amd Jones PA (1994) Mutational spectrum in p53 gene in bladder tumors from the endemic area of black foot disease in Taiwan. *Carcinogenesis* **15**: 1085–1087

Tseng CH, Chong CK, Chen CJ, Lin BJ and Tai TY (1995) Abnormal peripheral microcirculation in seemingly normal subjects living in blackfoot-disease-hyperendemic villages in Taiwan. *Int J Microcirc: Clin Exp* **15**: 21–27

Tseng WP, et al (1968) Prevalence of skin cancer in an endemic area of chronic arsenism in Taiwan. *J Natl Cancer I* **40**: 453

van Kranen HJ, de Gruijl FR, de Vries A, Sontag Y, Wester PW, Senden HC, Rozemuller E and van Kreijl CF (1995) Frequent p53 alterations but low incidence of ras mutations in UV-B-induced skin tumors of hairless mice. *Carcinogenesis* **16**: 1141–1147

van Kranen HJ, de Laat A, van de Ven J, Wester PW, de Vries A, Berg RJ, van Kreijl CF and de Gruijl FR (1997) Low incidence of p53 mutations in UVA (365-nm)-induced induced skin tumors in hairless mice. *Cancer Res* **57**: 1238–1240

Weyers W, Bonczkowitz M, Weyers I, Bittinger A and Schill W-B (1996) Melanoma in situ versus melanocytic hyperplasia in sun-damaged skin: assessment of the significance of histopathologic criteria for differential diagnosis. *Am J Dermatopathol* **18**: 560–566

Wu MM, Kuo TL, Hwang YH and Chen CJ (1989) Dose-response relation between arsenic concentration in well water and mortality from cancers and vascular diseases. *Am J Epidemiol* **130**: 1123–1132

Ziegler A, Leffell DJ and Kunala S (1993) Mutation hotspots due to sunlight in the p53 gene of nonmelanoma skin cancer. *Proc Natl Acad Sci USA* **90**: 4216–4220

Ziegler A, Jonason AS, Leffell DJ, Simon JA, Sharma HW, Kimmelman J, Remington L, Sachs T and Brash DE (1994) Sunburn and p53 in the onset of skin cancer. *Nature* **372**: 773–776

*© 1999 Cancer Research Campaign*

## References

[PDF_00402] Berg R. J., van Kranen H. J., Rebel H. G., de Vries A., van Vloten W. A., Van Kreijl C. F., van der Leun J. C., de Gruijl F. R. (1996). Early p53 alterations in mouse skin carcinogenesis by UVB radiation: immunohistochemical detection of mutant p53 protein in clusters of preneoplastic epidermal cells.. Proc Natl Acad Sci U S A.

[PDF_00407] Bishop J. M. (1991). Molecular themes in oncogenesis.. Cell.

[PDF_00411] Brown K. G., Chen C. J. (1995). Significance of exposure assessment to analysis of cancer risk from inorganic arsenic in drinking water in Taiwan.. Risk Anal.

[PDF_00414] Cavigelli M., Li W. W., Lin A., Su B., Yoshioka K., Karin M. (1996). The tumor promoter arsenite stimulates AP-1 activity by inhibiting a JNK phosphatase.. EMBO J.

[PDF_00417] Chen C. J., Wu M. M., Lee S. S., Wang J. D., Cheng S. H., Wu H. Y. (1988). Atherogenicity and carcinogenicity of high-arsenic artesian well water. Multiple risk factors and related malignant neoplasms of blackfoot disease.. Arteriosclerosis.

[PDF_00421] Chiang H. S., Guo H. R., Hong C. L., Lin S. M., Lee E. F. (1993). The incidence of bladder cancer in the black foot disease endemic area in Taiwan.. Br J Urol.

[PDF_00426] Chiou H. Y., Hsueh Y. M., Liaw K. F., Horng S. F., Chiang M. H., Pu Y. S., Lin J. S., Huang C. H., Chen C. J. (1995). Incidence of internal cancers and ingested inorganic arsenic: a seven-year follow-up study in Taiwan.. Cancer Res.

[PDF_00429] Debec A., Courgeon A. M., Maingourd M., Maisonhaute C. (1990). The response of the centrosome to heat shock and related stresses in a Drosophila cell line.. J Cell Sci.

[PDF_00432] Drouin R., Therrien J. P. (1997). UVB-induced cyclobutane pyrimidine dimer frequency correlates with skin cancer mutational hotspots in p53.. Photochem Photobiol.

[PDF_00435] Hsueh Y. M., Cheng G. S., Wu M. M., Yu H. S., Kuo T. L., Chen C. J. (1995). Multiple risk factors associated with arsenic-induced skin cancer: effects of chronic liver disease and malnutritional status.. Br J Cancer.

[PDF_00438] Hsueh Y. M., Huang Y. L., Huang C. C., Wu W. L., Chen H. M., Yang M. H., Lue L. C., Chen C. J. (1998). Urinary levels of inorganic and organic arsenic metabolites among residents in an arseniasis-hyperendemic area in Taiwan.. J Toxicol Environ Health A.

[PDF_00441] Inga A., Scott G., Monti P., Aprile A., Abbondandolo A., Burns P. A., Fronza G. (1998). Ultraviolet-light induced p53 mutational spectrum in yeast is indistinguishable from p53 mutations in human skin cancer.. Carcinogenesis.

[PDF_00445] Innis M. A., Myambo K. B., Gelfand D. H., Brow M. A. (1988). DNA sequencing with Thermus aquaticus DNA polymerase and direct sequencing of polymerase chain reaction-amplified DNA.. Proc Natl Acad Sci U S A.

[PDF_00451] Lee-Chen S. F., Gurr J. R., Lin I. B., Jan K. Y. (1993). Arsenite enhances DNA double-strand breaks and cell killing of methyl methanesulfonate-treated cells by inhibiting the excision of alkali-labile sites.. Mutat Res.

[PDF_00449] Lee T. C., Tanaka N., Lamb P. W., Gilmer T. M., Barrett J. C. (1988). Induction of gene amplification by arsenic.. Science.

[PDF_00454] Lin S. R., Lee Y. J., Tsai J. H. (1994). Mutations of the p53 gene in human functional adrenal neoplasms.. J Clin Endocrinol Metab.

[PDF_00458] Lynn S., Shiung J. N., Gurr J. R., Jan K. Y. (1998). Arsenite stimulates poly(ADP-ribosylation) by generation of nitric oxide.. Free Radic Biol Med.

[PDF_00456] Lübbe J., Kleihues P., Burg G. (1994). Das Tumorsuppressor-Gen p53 und seine Bedeutung für die Dermatologie.. Hautarzt.

[PDF_00460] Magos L. (1991). Epidemiological and experimental aspects of metal carcinogenesis: physicochemical properties, kinetics, and the active species.. Environ Health Perspect.

[PDF_00463] Matsumura Y., Nishigori C., Yagi T., Imamura S., Takebe H. (1996). Characterization of p53 gene mutations in basal-cell carcinomas: comparison between sun-exposed and less-exposed skin areas.. Int J Cancer.

[PDF_00471] Nagano T., Ueda M., Ichihashi M. (1993). Expression of p53 protein is an early event in ultraviolet light-induced cutaneous squamous cell carcinogenesis.. Arch Dermatol.

[PDF_00467] Nakazawa H., English D., Randell P. L., Nakazawa K., Martel N., Armstrong B. K., Yamasaki H. (1994). UV and skin cancer: specific p53 gene mutation in normal skin as a biologically relevant exposure measurement.. Proc Natl Acad Sci U S A.

[PDF_00474] Nataraj A. J., Black H. S., Ananthaswamy H. N. (1996). Signature p53 mutation at DNA cross-linking sites in 8-methoxypsoralen and ultraviolet A (PUVA)-induced murine skin cancers.. Proc Natl Acad Sci U S A.

[PDF_00477] Oram Y., Orengo I., Baer S. C., Ocal T. (1994). p53 Protein expression in squamous cell carcinomas from sun-exposed and non-sun-exposed sites.. J Am Acad Dermatol.

[PDF_00480] Pan T. C., Horng C. J., Lin S. R., Lin T. H., Huang C. W. (1993). Simultaneous determination of Zn, Cd, Pb, and Cu in urine of patients with blackfoot disease using anodic stripping voltammetry.. Biol Trace Elem Res.

[PDF_00485] Sato M., Nishigori C., Zghal M., Yagi T., Takebe H. (1993). Ultraviolet-specific mutations in p53 gene in skin tumors in xeroderma pigmentosum patients.. Cancer Res.

[PDF_00488] Shibata A., Ohneseit P. F., Tsai Y. C., Spruck C. H., Nichols P. W., Chiang H. S., Lai M. K., Jones P. A. (1994). Mutational spectrum in the p53 gene in bladder tumors from the endemic area of black foot disease in Taiwan.. Carcinogenesis.

[PDF_00491] Tseng C. H., Chong C. K., Chen C. J., Lin B. J., Tai T. Y. (1995). Abnormal peripheral microcirculation in seemingly normal subjects living in blackfoot-disease-hyperendemic villages in Taiwan.. Int J Microcirc Clin Exp.

[PDF_00494] Tseng W. P., Chu H. M., How S. W., Fong J. M., Lin C. S., Yeh S. (1968). Prevalence of skin cancer in an endemic area of chronic arsenicism in Taiwan.. J Natl Cancer Inst.

[PDF_00503] Weyers W., Bonczkowitz M., Weyers I., Bittinger A., Schill W. B. (1996). Melanoma in situ versus melanocytic hyperplasia in sun-damaged skin. Assessment of the significance of histopathologic criteria for differential diagnosis.. Am J Dermatopathol.

[PDF_00507] Wu M. M., Kuo T. L., Hwang Y. H., Chen C. J. (1989). Dose-response relation between arsenic concentration in well water and mortality from cancers and vascular diseases.. Am J Epidemiol.

[PDF_00512] Ziegler A., Jonason A. S., Leffell D. J., Simon J. A., Sharma H. W., Kimmelman J., Remington L., Jacks T., Brash D. E. (1994). Sunburn and p53 in the onset of skin cancer.. Nature.

[PDF_00510] Ziegler A., Leffell D. J., Kunala S., Sharma H. W., Gailani M., Simon J. A., Halperin A. J., Baden H. P., Shapiro P. E., Bale A. E. (1993). Mutation hotspots due to sunlight in the p53 gene of nonmelanoma skin cancers.. Proc Natl Acad Sci U S A.

[PDF_00496] van Kranen H. J., de Gruijl F. R., de Vries A., Sontag Y., Wester P. W., Senden H. C., Rozemuller E., van Kreijl C. F. (1995). Frequent p53 alterations but low incidence of ras mutations in UV-B-induced skin tumors of hairless mice.. Carcinogenesis.

[PDF_00500] van Kranen H. J., de Laat A., van de Ven J., Wester P. W., de Vries A., Berg R. J., van Kreijl C. F., de Gruijl F. R. (1997). Low incidence of p53 mutations in UVA (365-nm)-induced skin tumors in hairless mice.. Cancer Res.

